# Association Between Hypocomplementemia (C3 and C4) and MRI Findings in Different Neuropsychiatric Lupus Syndromes in a Tertiary Hospital

**DOI:** 10.7759/cureus.17939

**Published:** 2021-09-13

**Authors:** Yasser M Bawazir, Sami Bahlas, Ibtisam Jali, Deyaa A Mukhtar, Nujood Almohammmadi, Mohammad Mustafa

**Affiliations:** 1 Rheumatology/Internal Medicine, King Abdulaziz University Faculty of Medicine, Jeddah, SAU; 2 Medicine, King Abdulaziz University Faculty of Medicine, Jeddah, SAU; 3 Medicine, Hera General Hospital, Makkah, SAU; 4 Medicine, King Fahad Hospital, Al Madinah, SAU; 5 Rheumatology/Internal Medicine, University of Jeddah, Jeddah, SAU

**Keywords:** saudi arabia, complements, mri, npsle, sle

## Abstract

Objective

The aim of this study was to describe the magnetic resonance imaging (MRI) findings and correlate them with the complements level.

Methodology

This is a retrospective chart review study involving 187 lupus patients attending the rheumatology clinic during the period between 2010 and 2020. Out of the 187 patients, only 49 patients were diagnosed to have neuropsychiatric lupus manifestation and underwent MRI study.

Results

We included 49 neuropsychiatric systemic lupus erythematosus patients with a mean age of 35.33 years; most of them were Saudi (51%), with disease duration between -six and nine years (40.8%). In regard to MRI brain findings, 51% had abnormal findings, most commonly white matter changes in 42.9% followed by contrast enhancement in 36.7% and mild volume loss in 16.3%. Regarding the complement level, 21 (42.9%) patients had a low C3 level and 35 (71.4%) had a low C4 level. Lastly, following the main objective, C3 and C4 do not have a statistically significant relationship with white matter lesion given the sample of this data (p = 0.589 and p = 0.657, respectively).

Conclusion

MRI provides a significant clinical information to evaluate neuropsychiatric lupus manifestations. These clinical data can be correlated with immunological findings, which can help in the early diagnosis and management of this disease.

## Introduction

Systemic lupus erythematosus (SLE) is an autoimmune connective tissue disorder with various clinical presentations that affects many organ systems, including the central and peripheral nervous systems and muscles [1]. Neuropsychiatric SLE (NPSLE) is a life-threatening form of the disease; thus, early diagnosis and proper treatment are critical for the management of these patients. NPSLE can manifest with a range of neurological and psychiatric symptoms, as classified by the American College of Rheumatology (ACR) into 19 neuropsychiatric syndromes [2]. Approximately one-third of all NPSLE events in patients with SLE are primary manifestations of SLE-related autoimmunity, with seizure disorders, cerebrovascular disease, acute confusional state, and neuropathy being the most common [2].

Ahn et al. studied the possible risk factors for NPSLE in 1,121 patients with SLE of whom 429 developed NPSLE. They found that patients with a high SLE disease activity index, antiphospholipid antibody positivity, fewer years of education, and absence of anti-double strand deoxyribonucleic acid (anti-dsDNA) antibody at the time of diagnosis were at a higher risk of developing NPSLE [3].

The pathogenesis of NPSLE is poorly understood, and two theories have been proposed. Based on the blood-brain barrier (BBB) permeability theory, the BBB becomes more permeable to neuropathic antibodies, whereas the second theory suggests a defect in the blood-cerebrospinal fluid barrier in the choroid plexus, which allows access of neuropathic antibodies to the nervous system and development of immune complexes [4].

Defective clearing of apoptotic material contributes to the formation of autoantibodies and immune complexes in SLE [5]. As the complement system is an important host mechanism for the removal of atypical antigens and immune complexes, hypocomplementemia in SLE is considered a serological sign of impending or ongoing inflammation where complement factors are “consumed” by tissue-bound immune complexes [6-8].

Despite their inclusion in SLE disease activity indices and classification criteria [9-10], the relationship between hypocomplementemia (C3 and C4), neuropsychiatric syndromes, and magnetic resonance imaging (MRI) findings has not been systematically investigated. It is unknown whether hypocomplementemia predicts neuropsychiatric lupus activity. In a cohort of 2,399 patients with SLE, 55% had low C3 levels and 47% had low C4 levels, and neuropsychiatric manifestations, including seizure, psychosis, and stroke, were significantly associated with both low C3 and C4 levels [11].

In this study, we aimed to investigate the relationship among hypocomplementemia, MRI findings, and neuropsychiatric manifestations in patients with NPSLE and establish the relative importance of radiological imaging findings in different NPSLE syndromes.

## Materials and methods

Study design and patients

This was a retrospective chart review study conducted at King Abdulaziz University Hospital in Jeddah, Saudi Arabia. We identified patients diagnosed with SLE based on the ACR criteria who were treated at the Rheumatology Clinic between 2010 and 2020. Pediatric patients, those with overlap syndrome, and those who did not fulfill the ACR criteria were excluded from the study. Patients who were presented with central nervous system (CNS) manifestation and fulfilled the ACR criteria for the diagnosis of neuropsychiatric lupus were identified and selected.

The study was approved by the Ethics Committee of King Abdulaziz University Hospital.

Data collection

Data were collected from the electronic medical record system of the hospital. The following data were recorded: demographic characteristics (age, nationality, and marital status), clinical features (diabetes mellitus, hypertension, disease duration, stage of nephritis, and NPSLE manifestations such as cerebrovascular disease, epilepsy, and mental disorder), and laboratory data (proteinuria, anti-phospholipid antibody, anti-nuclear antibody [ANA], anti-dsDNA, and complement levels).

Brain MRI scans obtained within six months after symptom onset were also evaluated. Brain MRI was performed for all patients using a 1.5 Tesla (SIGNA) machine (General Electric, Boston, MA, USA) following the standard procedures. The following sequences were obtained: T1-weighted, T2-weighted, fluid-attenuated inversion recovery, and diffusion-weighted images. In some patients, gadolinium enhancement was performed on T1-weighted or fluid-attenuated inversion recovery images. The interpretation of the images was conducted by a neuroradiologist consultant at King Abdulaziz University Hospital.

Outcome measures

The primary outcome measures were the predictive role of immunological factors and their relationship with the brain MRI changes in NPSLE.

Statistical analysis

All data were analyzed using IBM SPSS Statistics for Windows, version 24 (IBM Corp., Armonk, NY, USA) and visually presented using GraphPad Prism, version 8 (GraphPad Software, Inc., San Diego, CA, USA). Descriptive statistics was used to analyze the characteristics of the study patients. Categorical and nominal variables were presented as counts and percentages, and continuous variables were presented as means and standard deviations. To determine the correlation of white matter lesions, infarction, and brain atrophy with demographic and other variables, we used the chi-square test for categorical variables and the independent t-test for continuous variables with normal distribution. Otherwise, Welch’s t-test was used instead of the independent t-test. Finally, a conventional p-value of less than 0.05 was used to reject the null hypothesis. A private biostatistician was consulted to review the results.

## Results

Of the identified 187 patients with SLE, only 49 (26.2%) were diagnosed with NPSLE based on the ACR criteria. Their mean age was 35.33±13.6 years, and 51% of them were Saudis, with a disease duration between six and nine years (40.8%) (Table 1).

**Table 1:** Patients’ demographic data.

Demographics	Number of patients	Minimum	Maximum	Mean	SD
Age	49	17	73	35.33	13.6
		Count		%	
Total		49		100.0	
Nationality	Saudi	25		51.0	
	Non-Saudi	24		49.0	
Disease duration	Less than 3 years	5		10.2	
	3-6 years	10		20.4	
	6-9 years	20		40.8	
	More than 9 years	14		28.6	
Marital status	Single	31		63.3	
	Married	18		36.7	

With regard to the brain MRI findings, 51% of patients had abnormal findings, with the most common being white matter lesions (42.9%), followed by contrast enhancement (36.7%) and mild volume loss (16.3%) (Table 2).

**Table 2:** MRI findings in the study sample. PRES, Posterior reversible encephalopathy syndrome

Variables		Number of patients	%
Total		49	100.0
MRI findings	Normal	24	49.0
	Abnormal	25	51.0
White matter lesions	Negative	28	57.1
	Positive	21	42.9
Mild volume loss	Negative	41	83.7
	Positive	8	16.3
Infarction	Negative	47	95.9
	Positive	2	4.1
Sagittal thrombosis	Negative	48	98.0
	Positive	1	2.0
Hemorrhage	Negative	48	98.0
	Positive	1	2.0
PRES	Negative	47	95.9
	Positive	2	4.1
Contrast enhancement	Negative	31	63.3
	Positive	18	36.7

ANA measured using the immunofluorescence technique was strongly positive in 73.5%, moderately positive in 20.4%, and mildly positive in 6.1% of patients. Regarding the anti-dsDNA status, 42 (85.7%) patients had a positive test with a high antibody level, and seven (14.3%) patients had a normal test. Moreover, 21 (42.9%) patients had low C3 levels and 35 (71.4%) had low C4 levels (Table 3). These levels were taken at the time of neurological symptoms.

**Table 3:** Autoantibody and complement levels in the study sample. Anti-dsDNA, antidouble-strand deoxyribonucleic acid antibody; CRP, C-reactive protein; ANA, anti-nuclear antibody; C3, complement 3’ C4, complement 4.

Variables	Number of patients	Minimum	Maximum	Mean	SD
Anti-dsDNA level (ref: 0-200 IU/mg)	49	42	2,166	752.86	592.9
CRP level (ref: <3.3 mg/dL)	23	2.90	17.40	7.08	4.4
		N		%	
Total		49		100.0	
ANA positivity	Mild (1/320)	3		6.1	
	Moderate (1/640)	10		20.4	
	Strong (1/1,280)	36		73.5	
Anti-dsDNA	Normal	7		14.3	
	Positive	42		85.7	
C3 level (ref: 0.75-1.65 g/L)	Low	21		42.9	
	Normal	24		49.0	
	High	1		2.0	
	N/A	3		6.1	
C4 level (ref: 0.2-0.6 g/L)	Low	35		71.4	
	Normal	11		22.4	
	N/A	3		6.1	

The most common NPSLE manifestation was headache (76.9%), followed by seizures (41%), stroke (15.4%), and psychosis (10.3%) (Table 4).

**Table 4:** Neuropsychiatric manifestations reported in the study sample.

Presentation	Number of patients	%
Total	49	100.0
Headache	30	76.9
Seizures	16	41.0
Stroke	6	15.4
Psychosis	4	10.3
Loss of consciousness	3	7.3
Lower limb weakness	1	2.6
Cranial nerve dysfunction	1	2.6
Vertigo	1	2.2

We identified 17 (34.7%) patients with proteinuria, and kidney biopsies were obtained from nine patients. The results showed class 3+5 nephritis in three patients, class 4 in one patient, and class 4+6 in one patient (Table 5). The majority of our patients did not have thromboembolic disease; however, two (4.1%) patients had deep venous thrombosis and only one (2%) patient had chronic pulmonary embolism. Regarding chronic diseases, diabetes mellitus was reported in four (8.2%) patients and hypertension in 19 (38.8%) patients (Table 6).

**Table 5:** Prevalence of lupus nephritis and antiphospholipid antibodies in the study sample. PT, prothrombin time; PTT, partial thromboplastin time

Variables		Count	%
Total		49	100.0
Proteinuria	Negative	32	65.3
	Positive	17	34.7
Nephritis stage	Not done	7	14.6
	Not indicated	29	60.4
	No indication for biopsy	2	4.2
	No biopsy results	1	2.1
	Mesangioproliferative	1	2.1
	Focal proliferative	1	2.1
	Diffuse proliferative	2	4.2
	Class 4+6	1	2.1
	Class 4	1	2.1
	Class 3+5	3	6.3
	Missing	1	
PT (ref: 11-14 seconds)	Normal	43	89.6
	High	2	4.2
	N/A	3	6.3
	Missing	1	
PTT (ref: 29-40 seconds)	Normal	38	79.2
	High	7	14.6
	N/A	3	6.3
	Missing	1	
Anti-cardiolipin IgM (ref: 0-12 IU/mL)	Negative	36	75.0
	Positive	5	10.4
	Equivocal	1	2.1
	Not done	6	12.5
	Missing	1	
Anti-cardiolipin IgG (ref: 0-12 IU/mL)	Negative	33	71.7
	Positive	3	6.5
	Not done	10	21.7
	Missing	3	
Lupus anticoagulants (ref: >45)	Negative	38	80.9
	Positive	4	8.5
	Not done	5	10.6
	Missing	2	
Beta 2 glycoprotein IgM (ref: (0-20 CU)	Negative	32	69.6
	Not done	14	30.4
	Missing	3	
Beta 2 glycoprotein IgG (ref: 0-20 CU)	Negative	32	68.1
	Not done	14	29.8
	Equivocal	1	2.1
	Missing	2	

**Table 6:** Distribution of thromboembolic diseases and chronic metabolic disorders in the study sample. DVT, deep venous thrombosis; PE, pulmonary embolism; DM, diabetes mellitus; LDL, low-density lipoprotein; HDL, high-density lipoprotein

Variables		Count	%
Total		49	100.0
DVT	Not present	43	87.8
	Present	2	4.1
	Sagittal venous thrombosis	2	4.1
	Mesenteric thrombosis	1	2.0
	Left femoral vein thrombosis	1	2.0
PE	Not present	48	98.0
	Chronic PE	1	2.0
Abortion	No	44	89.8
	Yes	1	2.0
	Unknown	4	8.2
DM	No	45	91.8
	Yes	4	8.2
Hypertension	No	30	61.2
	Yes	19	38.8
LDL (ref: 0-3.57 mmol/L)	Normal	7	50.0
	High	2	14.3
	N/A	5	35.7
	Missing	35	
HDL (ref: 0.9-1.55 mmol/L)	Low	2	15.4
	Normal	1	7.7
	High	4	30.8
	N/A	6	46.2
	Missing	36	

In the multivariable analysis to determine the correlation between the MRI findings and demographic and clinical variables (Tables 7, 8), white matter lesions were the only MRI variable with sufficient representation and distribution to be included in correlation and regression models. Generally, all results were non-significant. However, patients with white matter lesions had a higher anti-dsDNA titer (904.24 IU/mL) compared with those without white matter lesions (639.32 IU/mL), although the difference was not statistically significant. Furthermore, white matter lesions were more prevalent among patients with strong ANA positivity than in those with a negative test, also without statistical significance (p = 0.054). Finally, C3 and C4 levels did not show a statistically significant relationship with white matter lesions (p = 0.589 and p = 0.657, respectively). Since no independent variables were identified as significant prognostic factors in this study sample, a regression model was not necessary for further analysis.

**Table 7:** Relationship between demographic factors and white matter lesions.

Demographics		Number of patients	White matter lesions		p-Value
			Negative	Positive	
Age (years)		49	33.21±10.5	38.14±16.7	0.244
Nationality	Saudi	25	13 (52.0%)	12 (48.0%)	0.458
	Non-Saudi	24	15 (62.5%)	9 (37.5%)	
Disease duration	Less than 3 years	5	4 (80.0%)	1 (20.0%)	0.188
	3-6 years	10	8 (80.0%)	2 (20.0%)	
	6-9 years	20	10 (50.0%)	10 (50.0%)	
	More than 9 years	14	6 (42.9%)	8 (57.1%)	
Marital status	Single	31	18 (58.1%)	13 (41.9%)	0.864
	Married	18	10 (55.6%)	8 (44.4%)	

**Table 8:** Relationship between clinical variables and the incidence or prevalence of white matter lesions. Anti-dsDNA, anti-double strand DNA antibody; CRP, C-reactive protein; ANA, anti-nuclear antibody; C3, complement 3; C4, complement 4; LDL, low-density lipoprotein; HDL, high-density lipoprotein

Variables		Total	White matter lesions		p-Value
			Negative	Positive	
Anti-dsDNA level (ref: 0-200 IU/mg)		49	639.32±491.9	904.24±689.1	0.143
CRP level (ref: <3.3 mg/dL)		23	6.95±4.7	7.29±4.1	0.862
ANA positivity	Mild (1/320)	3	3 (100.0%)	0 (0.0%)	0.054
	Moderate (1/640)	10	8 (80.0%)	2 (20.0%)	
	Strong (1/1280)	36	17 (47.2%)	19 (52.8%)	
Anti-dsDNA	Normal	7	5 (71.4%)	2 (28.6%)	0.409
	Positive	42	23 (54.8%)	19 (45.2%)	
C3 level (ref: 0.75-1.65 g/L)	Low	21	10 (47.6%)	11 (52.4%)	0.589
	Normal	24	15 (62.5%)	9 (37.5%)	
	High	1	1 (100.0%)	0 (0.0%)	
	N/A	3	2 (66.7%)	1 (33.3%)	
C4 level (ref: 0.2-0.6 g/L)	Low	35	21 (60.0%)	14 (40.0%)	0.657
	Normal	11	5 (45.5%)	6 (54.5%)	
	N/A	3	2 (66.7%)	1 (33.3%)	
LDL (ref: 0-3.57 mmol/L)	Normal	7	4 (57.1%)	3 (42.9%)	0.155
	High	2	0 (0.0%)	2 (100.0%)	
	N/A	5	4 (80.0%)	1 (20.0%)	
HDL (ref: 0.9-1.55 mmol/L)	Low	2	0 (0.0%)	2 (100.0%)	0.202
	Normal	1	0 (0.0%)	1 (100.0%)	
	High	4	3 (75.0%)	1 (25.0%)	
	N/A	6	4 (66.7%)	2 (33.3%)	

## Discussion

In this study, we found no significant correlation of the brain MRI findings with the demographic and clinical variables, including complement levels, in patients with NPSLE, although white matter lesions were more prevalent among patients with strong ANA positivity and higher anti-dsDNA titers.

NPSLE has heterogeneous clinical syndromes, and definitive diagnosis is challenging without clinical assessment, laboratory, and neuroradiological tests. Brain MRI specifically reveals multiple brain lesions; however, there is no MRI abnormality that is pathognomonic for NPSLE. The most commonly reported lesions are small vessel disease, particularly white matter hyperintensities and lacunar infarcts, large vessel disease, inflammatory-like lesions (i.e., multifocal gray matter lesions), and brain atrophy [12]. In our study, approximately half of the patients had abnormal MRI findings, most commonly white matter lesions, followed by contrast enhancement and mild volume loss. These findings are consistent with those of previous studies, in which white matter changes and brain atrophy were found to be common among patients with SLE with neuropsychiatric manifestations compared with healthy controls [13].

The hypothesis that MRI abnormalities in NPSLE correspond to certain neuropsychiatric syndromes and immunological patterns introduced the possibility of novel diagnostic, prognostic, or treatment response biomarkers. Some observational studies have found an association between brain MRI abnormalities in NPSLE and low serum levels of C3 and C4 [14-15]. Liu et al. explored the effect of brain atrophy on NPSLE and found a relationship between hippocampal atrophy and the development of neuropsychiatric manifestations and lupus-related organ damage [16].

In a study enrolling 108 patients within six months of newly diagnosed NPSLE, Sarbu et al. found a statistically significant correlation of low complement and lupus anticoagulant levels with inflammatory-like lesions and white matter hyperintensities, respectively [17]. Magro-Checa et al., who also aimed to investigate the same hypothesis, found that several complement components were associated with NPSLE (C3 with diffuse manifestations and C4 with focal NPSLE), although only with the concomitant presence of antiphospholipid antibodies/disease activity [18].

In the present study, we found no statistically significant correlation between C3 and C4 levels and MRI findings; however, patients with NPSLE who had white matter lesions were found to have a higher prevalence of high anti-ds DNA and ANA titers compared with those without white matter lesions. These results may be explained with the small sample size. In our study population, the most common NPSLE manifestation was headache, followed by seizures, stroke, and psychosis, which is consistent with previously reported findings [19]. Hanly et al. found no association between headache and disease activity, medications, or autoantibodies, and the prevalence of headache associated with active disease was only 1.5% [20].

This study had several limitations, including the small sample size and the retrospective design. Furthermore, we did not include disease activity data using the international scoring systems, as well as data on the use of antiplatelet drugs, anticoagulants, and medications to treat lupus. There were missing data about the ANA patterns and the previous complements levels. Despite these limitations, this study reflects the most common CNS neuropsychiatric SLE manifestations and MRI findings in our population.

## Conclusions

MRI provides significant clinical information for the evaluation of NPSLE manifestations. These clinical data can be correlated with immunological findings, aiding the early diagnosis and management of this disease. Low complement level did not associate with brain MRI findings in our patients.
